# Concurrent environmental stressors and jellyfish stings impair caged European sea bass (*Dicentrarchus labrax*) physiological performances

**DOI:** 10.1038/srep27929

**Published:** 2016-06-15

**Authors:** Mar Bosch-Belmar, Folco Giomi, Alessandro Rinaldi, Alberta Mandich, Verónica Fuentes, Simone Mirto, Gianluca Sarà, Stefano Piraino

**Affiliations:** 1 Dipartimento di Scienze e Tecnologie Biologiche ed Ambientali (DiSTeBA), Università del Salento, Lecce, Italy; 2 Consorzio Nazionale Interuniversitario per le Scienze del Mare (CoNISMa), Rome, Italy; 3 Dipartimento di Scienze della Terra e del Mare (DISTEM), University of Palermo, Italy; 4 Istituto per l’Ambiente Marino Costiero, IAMC- CNR, Castellamare del Golfo (TP), Italy; 5 Dipartimento di Scienze della Terra, dell’Ambiente e della Vita (DISTAV), University of Genova, Italy; 6 Institut de Ciències del Mar, ICM-CSIC, Barcelona, Spain

## Abstract

The increasing frequency of jellyfish outbreaks in coastal areas has led to multiple ecological and socio-economic issues, including mass mortalities of farmed fish. We investigated the sensitivity of the European sea bass (*Dicentrarchus labrax*), a widely cultured fish in the Mediterranean Sea, to the combined stressors of temperature, hypoxia and stings from the jellyfish *Pelagia noctiluca,* through measurement of oxygen consumption rates (MO_2_), critical oxygen levels (PO_2crit_), and histological analysis of tissue damage. Higher levels of MO_2_, PO_2crit_ and gill damage in treated fish demonstrated that the synergy of environmental and biotic stressors dramatically impair farmed fish metabolic performances and increase their health vulnerability. As a corollary, in the current scenario of ocean warming, these findings suggest that the combined effects of recurrent hypoxic events and jellyfish blooms in coastal areas might also threaten wild fish populations.

Human activities are transforming coastal and marine ecosystems at local, regional, and global scales, exposing both individual organisms and biological communities to dramatic environmental changes by a complex array of interacting stressors^1^,^2^. The current trend of induced anthropogenic environmental changes includes increasing sea water temperatures, frequencies of hypoxia episodes, and ocean acidification^3^,^4^. Concurrently, zooplankton communities respond to anthropogenic- and climate-induced changes by strong variations in their spatial distribution, structure and function^5^,^6^. Jellyfish represent one of the components of plankton that seem to be responding positively to the ongoing changes. They are likely to affect the food web structure by their high consumption rates, fast growth and reproduction rates, and wide tolerance to ecosystem changes^7^,^8^,^9^. Recent analyses of jellyfish population dynamics in Mediterranean coastal zones suggested increasing abundance and frequency of bloom formation^10^,^11^,^12^,^13^. Global changes such as overfishing, eutrophication and ocean warming have been proposed as mechanisms leading to jellyfish increases in many coastal waters worldwide, including the Mediterranean Sea^7^,^11^,^14^,^15^,^16^. These factors are causing severe negative impacts on human economic activities, such as tourism, fisheries, and aquaculture^7^,^17^,^18^,^19^.

Aquaculture is an important source of income for local societies and sustains over 40% of global fish production; mariculture supports nearly 30% (US $23.5 billion) of the total value of farmed finfish species^20^. Interactions between jellyfish and marine caged fish have been recorded on several occasions in recent years, leading to severe fish mass mortality^21^. Jellyfish can enter fish cages either intact or fragmented, as tentacles and other body parts (e.g. oral arms), washed by currents and waves against the mesh of cage nets^21^,^22^,^23^. Overall, more than 400,000 salmon were killed in Irish marine aquaculture facilities in recurrent blooms of the scyphomedusa *Pelagia noctiluca* in 2007, 2013 and 2014^24^,^25^,^26^. The moon jellyfish *Aurelia aurita*, and the hydrozoans *Muggiaea atlantica* and *Phialella quadrata* were also involved in different farmed fish mortalities, and together with *P. noctiluca*, were identified as potentially harmful species for aquaculture facilities^22^. In addition, jellyfish can act as vectors of the bacterium *Tenacibaculum maritimum*, exacerbating fish gill injuries^27^. Beyond these few studies, limited information is available on how jellyfish affect fish health, the biological mechanisms underlying fish mortalities, or estimates of potential economic losses^21^. Only a few studies described significant fish injuries caused by the discharge of cnidocytes (specialized cnidarian stinging cells) in fish tissues (skin, gills) leading to envenomation and cellular damage^23^,^28^.

Temperature and dissolved oxygen concentration in the water column are crucial for the development and performance of aquatic organisms through direct effects on their metabolic rates^29^,^30^. Most fish adapt their physiological responses to sustain their metabolic rates when exposed to temperature changes or decreased dissolved oxygen levels^3^,^31^; however, additional external factors (such as pollutants or different environmental factor) may impair acclimation processes.

In this framework, we investigated the sensitivity of fish to the co-occurrence of environmental stressors (water temperature) and jellyfish stings to understand the impact of jellyfish blooms on caged finfish in a global warming scenario. Experiments were designed to test the combined effects of temperature (“temperature treatment”) and prolonged jellyfish contact (“jellyfish treatment”) on metabolic performances (MO_2_ and critical PO_2_) and tissue damage on fish gills over the time. We used the jellyfish *Pelagia noctiluca* (Forsskål, 1775), the strongest stinging and most abundant scyphozoan species in the Mediterranean Sea and Eastern North Atlantic, and juveniles of *Dicentrarchus labrax* (Linnaeus, 1758), one of the most common fish species in Mediterranean marine aquaculture. This study presents important eco-physiological data to the overall fish mariculture sector in jellyfish-affected coastal areas and also for the scientific community working on the global change susceptibility of wild fish populations.

## Results

### Histological analysis

**Figure 1:**
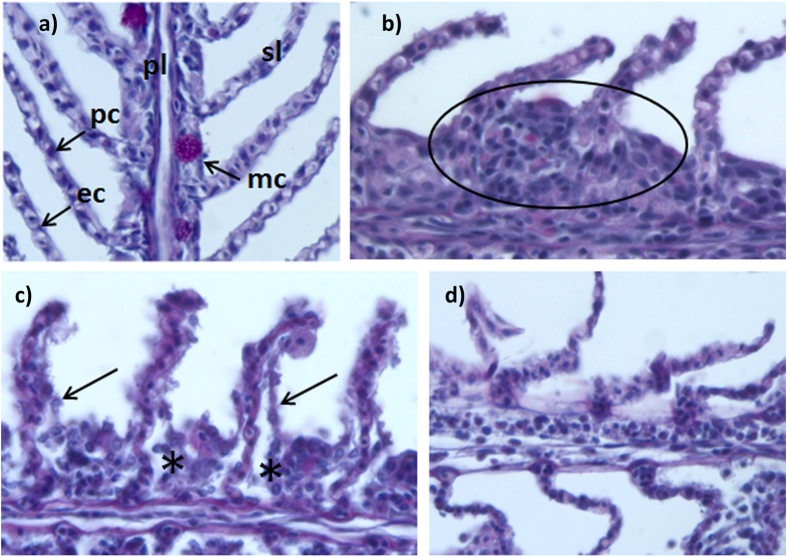
Gill lesions in fish exposed to *Pelagia noctiluca*. (**a**) Control fish gills with unaltered primary lamellae (pl) with mucous cells (mc) and elongated secondary lamellae (sl) with flat epithelial cells (ep) and pillar cells (pc), (400×); (**b–e**). Pathological features in fish gills from the treatment groups after 8 h exposure to jellyfish (400×): (**b**) Hyperplasia of primary lamella; (**c**) Moderate lifting of epithelial cells (*) and cellular degeneration (arrows); (**d**) Absence of respiratory epithelium and loss of structure.

**Figure 2:**
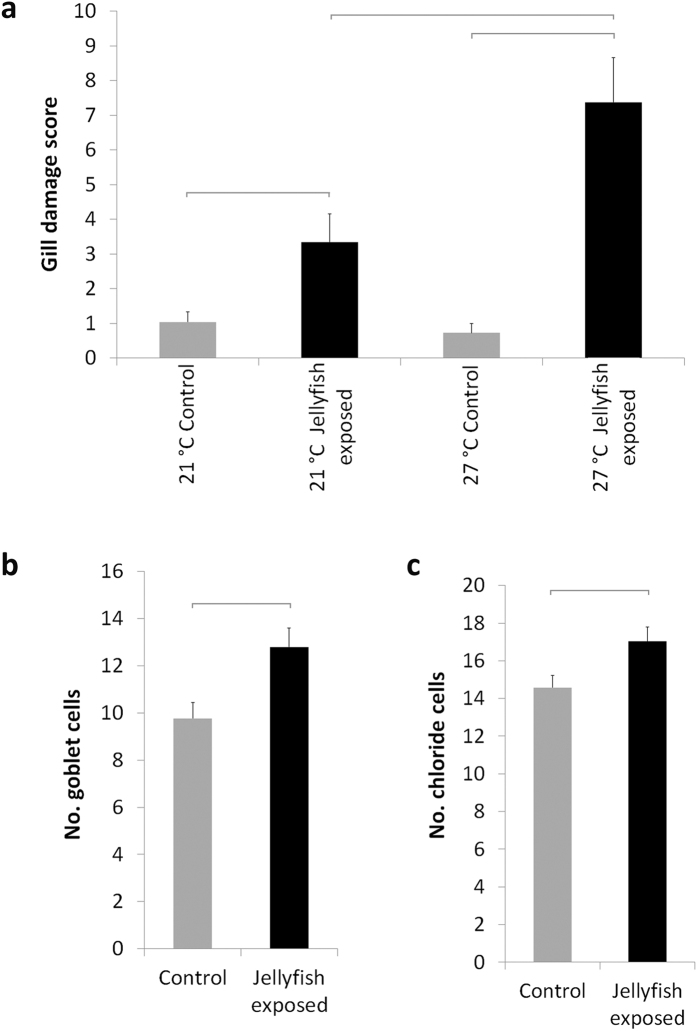
Gill damage scores (10-point scale) for each treatment (**a**), numbers of goblet cells (**b**) and chloride cells (**c**). Fish exposed to jellyfish (black bars) and control fish (grey bars). Horizontal grey lines indicate significant differences among treatments (p < 0.05), based on Kruskal-Wallis test for gill damage scoring and one-way ANOVAs for goblet and chloride cells.

The treatment groups showed obvious gill tissue injuries, most fish displaying lesions of clinical significance (Fig. 1). The most frequently observed cellular damages were hyperplasia and lamellar fusion, lamellar oedema and lifting, and cellular hypertrophy and degeneration especially in fish exposed to jellyfish at 27 °C. The gill damage scores in fish exposed to jellyfish were higher than in controls without jellyfish at both temperatures (21 and 27 °C) (Table 1, Fig. 2a). Wilcoxon pairwise comparisons showed significant interactions between temperature and jellyfish factors for treated fish but not for control groups (Table 2, Fig. 2a). The number of goblet cells was significantly higher in fish exposed to jellyfish than in controls; also, the number of chloride cells differed significantly between control and exposed fish, but not between control fish at different temperatures (Table 1, Fig. 2b,c).

### Respirometry measurements

The oxygen consumption rate that approximates the routine metabolism (MO_2_) of *D. labrax* juveniles was affected by jellyfish and temperature treatments (Table 3). In addition, statistically significant differences in the critical oxygen pressure (PO_2crit_) were found between control and jellyfish-treated fish, but not between temperatures. No significant changes were observed on fish MO_2_ and PO_2crit_ over time following their exposure to *P. noctiluca* tissues (Table 3).

**Figure 3:**
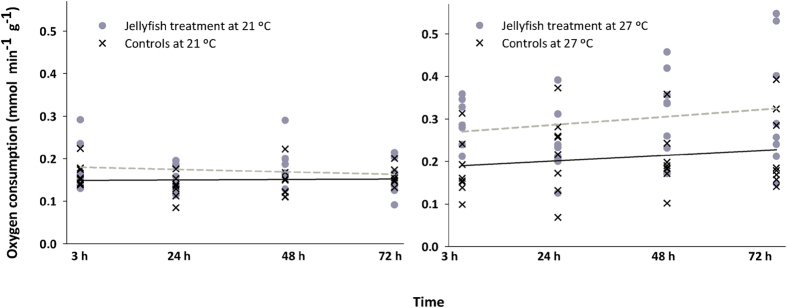
Oxygen consumption rates of *Dicentrarchus labrax* juveniles exposed to *Pelagia noctiluca* contact. Fish exposed to jellyfish are represented by grey dots and a dashed regression line; control fish are represented by black “x” and a continuous regression line. Experiments performed at 21 °C and 27 °C are shown on the left and right panels, respectively. X axes correspond to the time after the fish were exposed to jellyfish. Overall, oxygen consumption rates did not change over time: r^2^ is 0.02 for treated and 0.002 for controls at 21 °C (n.s.) and 0.04 for treated and 0.03 for controls at 27 °C (n.s.). Regression lines have equal intercepts at 21 °C; however, treated fish have higher oxygen consumption rates than controls at 27 °C; see Table 4 for significances.

**Figure 4:**
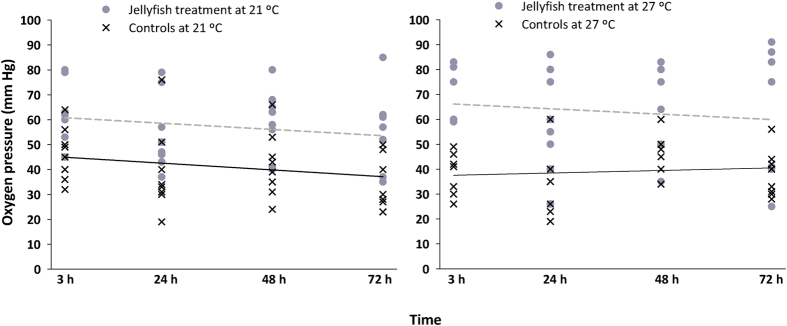
Critical oxygen pressures of *Dicentrarchus labrax* juveniles exposed to *Pelagia noctiluca* contact. Data from fish exposed to jellyfish are represented by grey dots and a dashed regression line; data from control fish are represented by black “x” and a continuous regression line. Experiments performed at 21 °C and 27 °C are shown on the left and right panels, respectively. X axes correspond to the time after the fish were exposed to jellyfish. PO_2crit_ did not change over time: r^2^ is 0.04 for treated and 0.05 for controls at 21 °C (n.s.) and 0.02 for treated and 0.01 for controls at 27 °C (n.s.). Regression lines have different intercepts at 21 °C and 27 °C showing higher PO_2crit _s for fish exposed to jellyfish.

**Figure 5:**
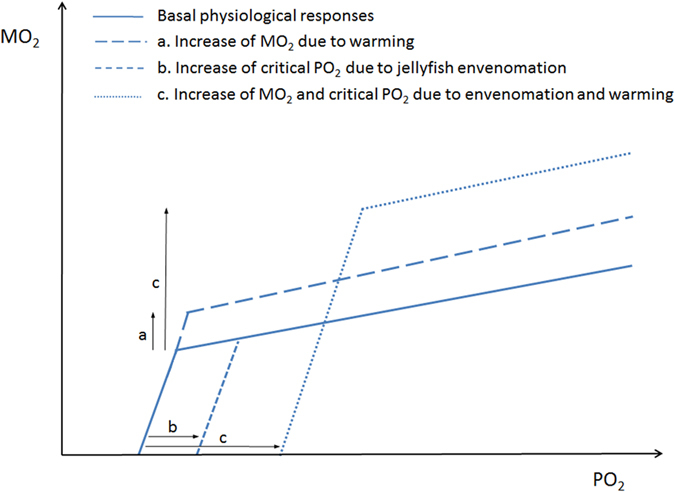
Unifying model of physiological responses of fish to the interaction of ocean warming and jellyfish stinging. Dashed lines represent the responses to single factors alone. Briefly, the rise of water temperature is mirrored by an increase of oxygen consumption rate (MO_2_), but does not affect the sensitivity of fish to declining environmental oxygen tension (PO_2_) (long dashed line); by contrast, jellyfish envenomation causes increased PO_2crit_, which enhances the sensitivity to hypoxia (short dashed line). The dotted line represents the physiological response to the interaction of both factors and shows the enhanced vulnerability of fish.

MO_2_ values were significantly different at the two temperatures (Table 4, Fig. 3). Oxygen uptake was equivalent in fish exposed to jellyfish stings and control fish at 21 °C, whereas fish exposed to jellyfish at 27 °C had higher MO_2_ than controls. PO_2crit_ at 21 °C and 27 °C were significantly different, with higher PO_2crit_ values in jellyfish-treated fish (averages ranged between 33–55 and 53–70 mm Hg, respectively) than in control fish (Table 3, Fig. 4). The observed changes of physiological responses of *D. labrax* juveniles (as MO_2_ and PO_2crit_ values) related to temperature and/or exposure to jellyfish tissues were represented in a conceptual model (Fig. 5). The higher temperature led to increased fish oxygen consumption rate (a), and the jellyfish stings produced an increased PO_2crit_ (b). The combined effects of temperature and jellyfish stings caused higher oxygen uptake and PO_2crit_ value (c) than the separate effect of either factor. The synergistic action of envenomation and increased temperature increased the PO_2crit_ value (i.e., anticipating the switch from the aerobic to anaerobic metabolism), thereby increasing fish sensitivity to hypoxic conditions.

## Discussion

Previous studies on jellyfish impacts on farmed fish hypothesized that respiratory distress may impair the overall fish metabolism^23^,^25^. Here, for the first time, we used an integrated approach to investigate the effects of jellyfish blooms on farmed fish by the combined analysis of fish gill integrity and metabolic rates. Significant effects (increased gill damage, oxygen consumption, and critical oxygen pressure) were observed in fish at higher temperature and exposed to jellyfish.

The increased histological damage in juvenile *D. labrax* exposed to *P. noctiluca* jellyfish corroborated previous observations of adult salmon (*Salmo salar*) with severe skin and gill injuries induced by jellyfish contacts, which significantly affected fish health and survival^23^,^25^. Severity of gill injuries increased with factors interaction (temperature and exposure to jellyfish), which reduced gill plasticity and functioning. The observed thickening of the gill epithelium due to hyperplasia may increase the diffusion distance for gas exchange, having profound effects on the efficiency of oxygen transfer across the gill^32^,^33^. The increase in goblet cell numbers in fish contacted by jellyfish was paralleled by [I] increased production of mucus (data not shown), which acts as a protective barrier against microbial infections^34^,^35^ but also forms a barrier to oxygen diffusion and contributes to hypoxia^36^, and [II] an increase of chloride cells in the respiratory epithelium, which is a common response to environmental (chemical or physical) stresses, such as low-calcium and low-magnesium water, or the detection of toxicants^37^,^38^.

With increasing temperature, metabolic rate and oxygen demand of ectothermic fish usually increase, but oxygen solubility declines, which exacerbates the problem caused by increased respiratory activity^2^. European sea bass increases cardiorespiratory and swimming performances in response to increased temperature^39^,^40^. Similarly, higher oxygen consumption rate^41^,^42^, growth rate, food intake and feeding efficiency^43^ also occur in higher temperature. Several studies indicate that temperature and hypoxia are likely to interact synergistically on fish metabolism^2^,^44^,^45^. PO_2crit_ values in fish usually increase when temperature rises^46^,^47^. Increased temperatures typically cause a decrease in the affinity of hemoglobin for oxygen, limiting oxygen uptake at the gills and, as a consequence, reducing fish tolerance to hypoxic conditions^2^,^31^. By contrast, other studies suggest that increased temperature may not affect the tolerance to hypoxia in some fish species due to the intervention of homeostatic mechanisms (e.g. the recruitment of tissue glycogen or liver lactate clearance capacity)^48^. An increase in PO_2crit_ values has been observed during digestion processes^49^ and may explain why hypoxic conditions might reduce appetite and growth in many fish species^49^,^50^. Increased PO_2crit_ values have also been observed after contamination by xenobiotics such as heavy metals, pesticides, or nanoparticles in coastal waters^51^,^52^.

As suggested by the conceptual model (Fig. 5), our results support the hypothesis that exposure to jellyfish stings and envenomation may act synergistically with temperature, reducing fish metabolic performance, impairing their ability to withstand hypoxic conditions and, as a consequence, reducing the available energy for critical processes such as growth and reproduction^2^. Furthermore, jellyfish venoms may have hemolytic properties^53^ leading to exacerbation of hypoxia. In conclusion, the interaction of jellyfish envenomations with increasing temperatures may result in greater vulnerability to hypoxic conditions and in the overall reduction of fish physiological performances.

The reduction of fish homeostatic potentials due to jellyfish outbreaks in coastal waters may co-occur to produce economic losses to aquaculture facilities. Our study suggests that the interaction of direct climatic stressors (e.g. warming) with indirect effects of global change (e.g. increasing jellyfish outbreaks) may exacerbate negative impacts on fish stocks. The consequences of such interactions for human activities are numerous, but mainly affect fisheries and aquaculture. Due to the continual growth of the aquaculture sector and the increased frequency of jellyfish blooms in coastal areas, the negative interactions of stinging jellyfish on farmed fish is expected to become a substantial, recurrent issue. More research on the effects of multiple stressors on fish populations in a global change scenario is needed for a better management of living resources and the development of effective mitigation plans.

## Materials and Methods

### Ethical statement

The study was performed in accordance with the EU Directive 2010/63 and Italian DL 2014/26; the experimental protocol was approved by the University of Palermo. Maintenance and handling of animals during the experiment, as well as the euthanasia procedure, were monitored and carried out by authorized staff to minimise the animals’ suffering.

### Animal collection and maintenance

Two hundred juvenile *Dicentrarchus labrax* (19.5 ± 5.5 g, means ± S.E.) were obtained from an aquaculture facility near Licata (Sicily, Italy). The choice of juveniles was related to severe mortality events caused by jellyfish in different Mediterranean aquaculture facilities where the most affected fish class ranged 15–60 g in weight^28^.

The fish were kept in tanks with seawater from a closed recirculated seawater system at controlled salinity, temperature and photoperiod (means ± S.E., 37.8 ±  0.08 salinity, 19.4 ± 0.4 °C, 12 h: 12 h light-dark regime). Acclimation at the experimental temperatures (21 °C and 27 °C) was gradually achieved during the week before the start of the experiments. The fish were fed daily with 2.5% of their body mass of commercial fish feed during the acclimation period. For the duration of the experiments, the fish stock density was maintained between 12.5 and 14 kg m^−3^, as used in *D. labrax* aquaculture cages (9–15 kg m^−3^). Jellyfish were collected by hand net from the port of Messina (Sicily, Italy) the day before the experiments and were maintained in 25-L buckets with filtered seawater and at low density (5 jellyfish per bucket) for one day.

### Experimental setup

The experiment was carried out at two temperatures, 21 °C and 27 °C. A total of 128 fish (64 treated, 64 controls) were subject to metabolic measurements. Fish were transferred to the treatment tanks 24 h prior to the start of the experiment and maintained unfed to reduce possible anomalous metabolic responses due to residual specific dynamic action. Sixteen 7-L treatment tanks were used for the 8-h contact period, each of them containing five fish to maintain the experimental stock density. The contact duration corresponds to a realistic night time period of high jellyfish concentration in surface waters^16^,^54^.

To simulate a realistic encounter between caged fish and jellyfish pressed by currents through aquaculture cages, jellyfish tissues were manually cut in small pieces (≥1 cm) immediately prior to the start of the jellyfish exposure. The jellyfish density used was 25 medusae m^−3^ 23. Tanks were supplied with air to keep dissolved oxygen at maximum levels and ensure contact between jellyfish pieces and fish. The treatment started when jellyfish tissues were randomly placed in 8 of 16 tanks with fish, whereas the other 8 tanks served as controls without jellyfish. Immediately after the exposure period at each temperature, all replicate fish groups were pooled into two 60-L tanks, according to their experimental status (treated or control) to maximise randomisation of subsequent fish sampling for metabolic measurements. At each of four different sampling times (3, 24, 48 and 72 h after the end of the contact period), 8 fish (4 control and 4 treated) were individually transferred into the respirometric chambers for acclimation. We opted to use closed respirometric chambers rather than swim tunnels to allow fish routine activity and spontaneous movement in a confined environment. We postulate this approach would fairly reflect the routine metabolic rate of cultured fish at high density and constrained living space conditions, such as in farming cage systems.

### Respirometry and determination of critical oxygen pressure (PO_2crit_)

At each temperature (21 and 27 °C), eight independent 2-L closed respirometers supplied with filtered sea water (Millipore GF/C 0.45 μm) were used to measure the oxygen consumption rate of individual fish. Chambers were covered with an opaque plastic material to avoid visual stresses to fish throughout measurements. An agitator and small magnets were used to maintain homogeneous water mixing inside the experimental chambers. At each sampling time (3, 24, 48 and 72 h after the end of jellyfish exposure), four treated and four control fish were randomly sampled and placed in individual respirometers. Fish were left undisturbed in the respirometers for 3 h with supplemented air to keep dissolved oxygen at the saturation level. Then the aerators were removed and chambers were carefully refilled of water and hermetically closed. Fibre-optic oxygen meters calibrated according to instructions by Pyro Science (Aachen, Germany) were used to record water oxygen levels. Fish were maintained in the respirometric chambers until the slope of the oxygen concentration curve changed suddenly. In most cases, that change occurred at 5 to 30% of the initial oxygen concentration. At the lowest oxygen concentration fish status was surely affected but no mortalities were recorded over the complete duration of the experiment. Fish were then removed from the respirometers, marked by a small cut in the caudal pin and returned to the original tank in order to maintain the initial density. All fish recovered well after hypoxic exposure.

Oxygen consumption rates to approximate routine metabolism (MO_2_) were calculated from the decrease in oxygen content in the respirometers over time and expressed as mmol min^−1^ g^−1^. Those times were standardized to 45 min at 21 °C and 15 min at 27 °C within the range during which the fish were able to oxyregulate. The critical oxygen pressure (PO_2crit_), which represents the transition from oxyregulation to oxyconformation during the progressive decline of environmental oxygen tension^30^, was calculated as the break-point of the graph depicting the PO_2_  − MO_2_ relationship. PO_2crit_ was expressed in mm Hg.

### Histological analysis of gill tissue

The experiments were performed in full compliance with the national rules (D.Lgs 116/92 and subsequent amendments) and the European Commission Recommendation guidelines for the accommodation and care of animals used for experimental and other scientific purposes (2007/526/EC). After the last sampling time (72 h), 16 experimental fish (4 controls and 4 treated at each temperature) were anaesthetised with 0.05% w/v MS222 (3-aminobenzoic acid ethyl ester) and then killed according to the current animal care rules using a lethal dose of MS-222 (0.1% w/v). Two gill arches were excised from each fish and immediately preserved in 10% neutral buffered formalin for 48 h and transferred to 70% ethanol for histological analysis. After dehydration, tissues were embedded in Paraplast (Bio-Optica), cut by microtome into 5 μm sections and stained using Hematoxylin-Eosin as “routine-staining” to reveal the underlying tissue structures and conditions. Moreover, the Periodic Acid Schiff (PAS) technique was used to identify the goblet cells. The localisation and the number of chloride cells was determined by immunocytochemical techniques by using a primary antibody that recognised sodium, potassium and chloride cotransporters (Na^+^/K^+^/Cl^−^ cotransporters NKCC1-T4 1:200) revealed by a second antibody Donkey Anti-Rabbit IgG (H+L) Alexa Fluor 488 (AF488) Conjugate (Southern Biotech), 1:200. For each gill arch, 9 randomly selected tissue areas, between 25 and 34 μm^2^ were screened at 200X and 400X to count the number of chloride cells.

### Gill damage score

Interpretation of the gill damage was based on a recently developed gill histopathology scoring system (GHS index, Mandich *et al*. in prep.) that rates the damage on each gill sample by a total score obtained by summation of partial scores assigned to 12 different criteria. The evaluation of gill damage was performed as follows: for each gill sample a total of 9 sections (photographed fields), each with 10 secondary lamellae were evaluated for all 12 histopathological criteria. For each criterion, the score ranged from 0 to 6 depending on the extent and intensity of injuries (0: no significant damage, 1: damage in 1–2 of 10 lamellae; 3: damage in 3–5 of 10 lamellae; 6: 6–10 of 10 lamellae damaged). Gill damage could be of different grades of severity, and advanced gill damage could mask previous mild injuries. Therefore, the GHS index was supplemented by a secondary classification system to separate different stages in the progression of tissue damage (according to Santos *et al*.^55^). All histopathological criteria were split into 3 groups. The first group (first-stage lesions) was composed of hyperplasia (cell number increase) of the lamina and the secondary lamellae, lamellar fusion, reduction of the lamellae, lamellar oedema (accumulation of an excessive amount of watery fluid in the intercellular spaces), and cellular hypertrophy (cell size increase). The second-stage lesions included circulatory disturbances of the lamina such as telangiectasia (dilation of groups of capillaries) and grave cellular anomalies (presence and extension of lamellar lifting); these more severe injuries lead to effects on tissue functions; the third -stage lesions included the appearance of haemorrhage, high granulocyte concentrations, and cellular degeneration of the respiratory epithelium or necrosis, which represent irreparable damages. The score assigned for each criterion was multiplied according to the severity group (×1: mild damage group; ×10: moderate injury group; and ×100: the most severe gill damage group).

The goblet and chloride cells were visually counted in each section and analysed separately from the other histopathological criteria.

### Statistical analysis

To obtain critical oxygen pressures (PO_2crit_) and approximate the break-point in the respiration curve, a Piecewise linear regression function was used (SigmaPlot v.11).

Normality of respiration data was confirmed with a Shapiro-Wilk test. To test the statistical significance among treatments for MO_2_ and for PO_2crit_, ANCOVA analyses were used, considering MO_2_ and PO_2crit_ as the *response* variables, time after the exposure period as a *continuous* explanatory variable, and temperatures and jellyfish treatments as *categorical* explanatory variables.

The assumptions of normality were not encountered for the histopathological data (Shapiro-Wilk test, p < 0.05). One-way Kruskal-Wallis test was performed to test the statistical significance among jellyfish-exposed and control fish at each temperature. Significant results were further tested by pairwise post-hoc comparisons (Wilcoxon test) adjusted for type I error. Differences in goblet cells and chloride cells were analysed using one-way analysis of variance (ANOVA).

The statistical software R (R Core Team 2015, v.3.2.2) was used to perform all analyses. Package (“coin”) was used to perform the Wilcoxon test^56^.

## Additional Information

**How to cite this article**: Bosch-Belmar, M. *et al*. Concurrent environmental stressors and jellyfish stings impair caged European sea bass (*Dicentrarchus labrax*) physiological performances. *Sci. Rep.* **6**, 27929; doi: 10.1038/srep27929 (2016).

## Figures and Tables

**Table 1:** Statistical results for histopathological gill damage.

Factor	Gill damage			Goblet cells			Chloride cells		
	df	H value	P value	df	F value	P value	df	F value	P value
Jellyfish	1	50.651	1.103 e^−12^	1	18.869	0.001	1	6.510	0.015
Temperature	1	0.010	0.919	1	3.996	0.046	1	3.580	0.063
Jelly. x Temp.		–		1	0.930	0.334	1	3.846	0.062

Kruskal-Wallis test for temperature and jellyfish factors and one-way ANOVA analyses for goblet cells and chloride cells. p < 0.05 was considered significant.

**Table 2:** Pair-wise comparisons for histopathological gill damage among temperature (21 and 27 °C) and jellyfish (Jelly.) treatments.

Gill damage	Jelly. x 21 °C	Controls x 21 °C	Jelly. x 27 °C	Controls x 27 °C
Jelly. x 21 °C	–	**<0.0005**	**0.0007**	**<0.0005**
Controls x 21 °C	51.0	–	**<0.0005**	0.1869
Jelly. x 27 °C	117.0	21.0	–	**<0.0005**
Controls x 27 °C	636.0	417.5	24.0	–

The F-values (lower left) and the p-values (upper right) are reported. Because multiple comparisons were performed, the Bonferroni’s correction was applied to the p-value (0.05/6 = 0.0083) and significant results are in bold.

**Table 3:** ANCOVA statistics for oxygen consumption rates (MO_2_) and critical oxygen levels (PO_2 crit_) of fish exposed to different temperatures (21 and 27 °C) and exposed or not to jellyfish.

Factor	MO_2_			PO_2 crit._		
	df	F value	P value	df	F value	P value
Time	1	1.357	0.246	1	2.349	0.128
Jellyfish	1	19.231	2.46 e^−05^	1	46.172	4.13 e^−10^
Temperature	1	53.849	2.56 e^−11^	1	0.173	0.678
Jelly. x Temp.	1	7.230	0.008	1	3.156	0.078

P < 0.05 was considered significant.

**Table 4:** Pair-wise comparisons for oxygen consumption rates (MO_2_) among temperature (21 °C and 27 °C) and jellyfish (Jelly.) factors.

MO2	Jelly. x 21 °C	Control x 21 °C	Jelly. x 27 °C	Control x 27 °C
Jelly. x 21 °C	–	0.0354	**<0.0005**	0.0299
Control x 21 °C	4.63	–	**<0.0005**	**<0.0005**
Jelly. x 27 °C	39.75	60.83	–	**<0.0005**
Control x 27 °C	4.95	14.38	15.03	–

The F-values (lower left) and the p-value (upper right) are reported. Because multiple comparisons were performed, the Bonferroni’s correction was applied to the p-value (0.05/6 = 0.0083) and significant results are in bold.
